# Pulmonary Embolism Complicating Active Pulmonary Tuberculosis: Two Case Reports of Tuberculosis-Associated Hypercoagulability

**DOI:** 10.3390/diagnostics16142140

**Published:** 2026-07-08

**Authors:** Denisa Maria Mitroi, Ramona Cioboata, Mihai Olteanu, Oana Maria Catana, Anca Lelia Riza, Viorel Biciusca

**Affiliations:** 1 Doctoral School, University of Medicine and Pharmacy of Craiova, 200349 Craiova, Romania; denisa_maria2@yahoo.com (D.M.M.); oana_cattana@yahoo.com (O.M.C.); 2 Department of Pneumology, University of Medicine and Pharmacy of Craiova, 200349 Craiova, Romania; biciuscaviorel@gmail.com; 3 Laboratory of Human Genomics, University of Medicine and Pharmacy of Craiova, 200638 Craiova, Romania; anca.costache@umfcv.ro

**Keywords:** tuberculosis, pulmonary embolism, hypercoagulability, venous thromboembolism, protein C, protein S, anticoagulation

## Abstract

**Background:** Active tuberculosis (TB) is increasingly recognized as a systemic thrombo-inflammatory condition capable of inducing a clinically relevant hypercoagulable state and increasing the risk of venous thromboembolism, including pulmonary embolism (PE). However, this association remains underrecognized in clinical practice, and its biological and therapeutic implications are not yet fully defined. We report two cases of active pulmonary tuberculosis complicated by PE and review the literature to highlight the temporal patterns, laboratory features, and clinical relevance of TB-associated hypercoagulability. **Case Presentation:** The first case involved a 65-year-old man with stage II chronic obstructive pulmonary disease in whom PE was identified concurrently with the diagnosis of active pulmonary TB. The second case concerned a 43-year-old man with severe pulmonary tuberculosis and subsequent intestinal involvement, in whom bilateral PE developed during the early intensive phase of antituberculous therapy. In both patients, laboratory evaluation demonstrated a consistent prothrombotic profile characterized by reactive thrombocytosis, elevated inflammatory markers, increased fibrinogen and D-dimer levels, and reduced protein C and protein S activity. Both patients received standard antituberculous therapy combined with therapeutic anticoagulation, with favorable clinical, laboratory, and radiological outcomes. **Discussion:** These cases are consistent with emerging evidence that active tuberculosis may induce a reversible infection-related hypercoagulable state through systemic inflammation, endothelial dysfunction, platelet activation, impaired fibrinolysis, and transient depletion of natural anticoagulants. They illustrate two clinically relevant temporal patterns described in the literature, namely PE detected at diagnosis and PE developing during early treatment despite appropriate therapy. The normalization of coagulation abnormalities after treatment further supports an acquired TB-related thrombo-inflammatory mechanism. **Conclusions:** Active tuberculosis may be complicated by pulmonary embolism both at presentation and during the early phase of treatment. Reactive thrombocytosis, elevated inflammatory markers, increased D-dimer levels, and reduced protein C and protein S activity may serve as useful indicators of TB-associated hypercoagulability. Pulmonary embolism should be considered in patients with severe tuberculosis who show unexplained deterioration or delayed recovery. Larger prospective studies are needed to clarify the role of coagulation profiling and biomarker-guided management in this setting.

## 1. Introduction

Tuberculosis (TB) is increasingly recognized not only as an infectious pulmonary disease but also as a systemic inflammatory and prothrombotic disorder. Growing epidemiological evidence indicates that active TB substantially increases the risk of venous thromboembolism (VTE), particularly pulmonary embolism (PE), with reported rates significantly higher than those observed in non-TB populations [1,2]. Meta-analytic data suggest that patients with active tuberculosis have nearly a threefold increased risk of VTE, while hospital-based cohorts further demonstrate that thromboembolic complications are associated with markedly increased mortality [1]. The pathophysiological basis of this association is complex and multifactorial. Active TB triggers an intense cytokine-mediated inflammatory response dominated by interleukin-1 (IL-1), interleukin-6 (IL-6), and tumor necrosis factor-alpha (TNF-α), which collectively activate coagulation pathways, increase fibrinogen and factor VIII synthesis, enhance plasminogen activator inhibitor-1 (PAI-1), and suppress endogenous anticoagulant mechanisms, including protein C and protein S [3,4,5]. This results in a hypercoagulable and hypofibrinolytic state that strongly predisposes patients to thrombus formation [6].

In parallel, endothelial activation and tissue factor expression promote thrombin generation and localized immunothrombosis, while reactive thrombocytosis and platelet hyperreactivity further amplify coagulation through platelet–monocyte crosstalk and release of mediators such as PF4, VEGF-A, and PDGF-BB. Importantly, multiple studies have shown that these abnormalities, particularly the reduction in natural anticoagulant proteins, tend to normalize after effective antituberculous therapy, supporting the concept of a reversible infection-driven thrombophilic state [7,8]. These data support the concept that severe active TB should be viewed as a multisystem thrombo-inflammatory condition, in which systemic inflammation, endothelial dysfunction, impaired fibrinolysis, platelet activation, and transient anticoagulant depletion converge to markedly increase thrombotic risk [9].

Against this background, we present two cases of active pulmonary tuberculosis complicated by pulmonary embolism that illustrate distinct clinical scenarios: pulmonary embolism identified at the time of tuberculosis diagnosis and pulmonary embolism developing during the early intensive phase of antituberculous treatment in severe multisystem disease. Together, these cases highlight the possible persistence of thrombotic risk during early treatment and the reversible nature of tuberculosis-associated coagulation abnormalities. Although the association between active tuberculosis and venous thromboembolism has been previously reported, the diagnostic challenge remains clinically relevant because pulmonary embolism may occur either at presentation or during early antituberculous therapy, when respiratory symptoms may be attributed to tuberculosis itself. The purpose of this report is therefore not to present TB-associated pulmonary embolism as a new entity but to illustrate two distinct temporal patterns and to emphasize the potential diagnostic value of persistent inflammation, rising D-dimer levels, reactive thrombocytosis, and transient reduction in protein C and protein S activity in patients with active tuberculosis.

## 2. Case Presentations

### 2.1. Case 1

A 65-year-old retired factory worker with a known history of stage II chronic obstructive pulmonary disease (COPD) presented with progressive worsening of his baseline respiratory symptoms over approximately three weeks. He reported a persistent and increasingly productive cough, occasionally associated with hemoptysis, progressive dyspnea on minimal exertion, intermittent fever, and profound fatigue that significantly limited his daily activities.

On admission, the patient was tachypneic with shallow respirations and oxygen saturation of 92% on room air. Pulmonary auscultation revealed newly developed crackles over the right upper lung field, raising suspicion for active parenchymal pathology.

Laboratory evaluation demonstrated a pronounced inflammatory and prothrombotic profile: CRP 120 mg/L, ESR 70 mm/h, reactive thrombocytosis (620,000/μL), normocytic anemia (hemoglobin 10.5 g/dL), and D-dimer markedly elevated at 970 ng/mL. Extended coagulation studies revealed protein C activity reduced to 50% and protein S activity reduced to 45%, collectively consistent with an acquired hypercoagulable state (Table 1). HIV serology was negative.

Contrast-enhanced chest CT demonstrated multiple centrilobular nodules and bilateral upper-lobe cavitary lesions (Figure 1), highly suggestive of active pulmonary tuberculosis. CT pulmonary angiography additionally revealed a 7 mm thrombus occluding a segmental branch of the right lower pulmonary artery, confirming concurrent acute pulmonary embolism (Figure 2).

Pulmonary tuberculosis was confirmed microbiologically by positive sputum smear microscopy for acid-fast bacilli, with molecular testing (GeneXpert) confirming Mycobacterium tuberculosis infection.

The patient was initiated on a standard four-drug antituberculous regimen including isoniazid, rifampicin, pyrazinamide, and ethambutol in accordance with National Tuberculosis Program guidelines together with therapeutic anticoagulation using low-molecular-weight heparin during the initial phase of treatment. He was subsequently transitioned to a novel oral anticoagulant, apixaban 2.5 mg twice daily, with close clinical and laboratory monitoring maintained throughout. Clinical evolution was favorable, with progressive resolution of fever, cough, and dyspnea, alongside normalization of inflammatory markers and coagulation parameters. Anticoagulation was continued with apixaban 2.5 mg twice daily, selected after consideration of potential drug–drug interactions with rifampicin and the patient’s overall thrombotic and bleeding risk; the total planned duration of anticoagulation was 6 months. At six-month follow-up, sputum microscopy was negative, and radiological reassessment confirmed significant regression of pulmonary lesions with complete resolution of the thromboembolic burden.

### 2.2. Case 2

A 43-year-old man, an active smoker with an 18 pack-year history and documented household contact with a tuberculosis case, presented for evaluation of progressive respiratory and constitutional symptoms evolving over two months. He reported productive cough, persistent drenching night sweats, and severe involuntary weight loss of approximately 15 kg, resulting in a markedly cachectic appearance at admission (body weight 57 kg). Physical examination revealed bilateral basal crackles and mild bilateral ankle edema, while initial hemodynamic parameters remained stable.

Laboratory evaluation demonstrated a severe inflammatory and prothrombotic profile: marked reactive thrombocytosis (750,000/μL), normocytic normochromic anemia (hemoglobin 9.8 g/dL), CRP 99 mg/L, ESR 90 mm/h, elevated fibrinogen (890 mg/dL), and D-dimer significantly elevated at 2500 ng/mL. Extended coagulation studies showed markedly reduced protein C activity (48%) and protein S activity (42%), alongside severe hypoalbuminemia (albumin 2.5 g/dL), findings collectively consistent with acquired TB-related hypercoagulability (Table 1). HIV serology was negative. Pulmonary tuberculosis was confirmed microbiologically by positive sputum smear microscopy (AFB 2+) and GeneXpert detection of Mycobacterium tuberculosis.

Chest CT demonstrated diffuse bilateral micronodules with a miliary distribution throughout both lungs, numerous tree-in-bud micronodules with areas of confluent consolidation associated with bronchial ectasia and internal microcalcifications predominantly involving the left upper lobe and the superior segment of the left lower lobe, and multiple cavitary lesions within the left upper lobe, the largest measuring 38 × 30 mm (Figure 3).

Standard first-line antituberculous therapy, consisting of isoniazid, rifampicin, pyrazinamide, and ethambutol, was promptly initiated in accordance with the National Tuberculosis Program guidelines. Despite appropriate treatment, the patient remained markedly inflammatory by day 15, with a significant rise in D-dimer levels. CT pulmonary angiography subsequently demonstrated bilateral pulmonary embolism, with thrombi measuring 6.6 mm on the right and 4 mm on the left, extending to the lobar and segmental branches (Figure 4). Therapeutic low-molecular-weight heparin was initiated immediately. He was subsequently transitioned to a novel oral anticoagulant, apixaban 2.5 mg twice daily, with close clinical and laboratory monitoring maintained throughout. Anticoagulation was continued with apixaban 2.5 mg twice daily for a planned duration of 12 months, in view of the extensive form of tuberculosis, the sustained inflammatory burden, and the patient’s overall thrombotic risk.

Twelve days later, the patient developed acute abdominal pain, hypotension, and clinical signs of acute abdomen. Thoracoabdominal CT revealed bowel distension, circumferential wall thickening, pneumatosis intestinalis, and moderate free intraperitoneal fluid, raising concern for intestinal ischemia in the context of ongoing TB-associated systemic inflammation. Emergency exploratory surgery identified multiple caseous intestinal masses adherent to bowel loops (Figure 5), and an ileostomy was performed. Histopathological examination confirmed multiple epithelioid granulomas with caseous necrosis, consistent with intestinal tuberculosis.

Postoperatively, the patient required intensive care support while continuing both full-dose antituberculous therapy and therapeutic anticoagulation. Clinical evolution was favorable, with gradual normalization of inflammatory markers, restoration of protein C and protein S activity to normal ranges, progressive nutritional recovery, and subsequent ileostomy closure. At six-month follow-up, sputum microscopy was negative, all laboratory parameters had normalized, and follow-up chest CT confirmed complete resolution of pulmonary emboli with marked regression of pulmonary lesions (Figure 6).

This case illustrates the multisystemic thrombo-inflammatory potential of severe active tuberculosis, where uncontrolled infection simultaneously drove pulmonary embolism and intestinal involvement, and highlights the reversibility of the acquired hypercoagulable state following effective combined therapy. It is noteworthy that the six-month follow-up evaluation in both patients coincided with the completion of the standard antituberculous treatment course (2HRZE/4HR), at which point full normalization of all coagulation parameters was documented.

## 3. Discussion and Review of the Literature

### 3.1. How Often Tuberculosis and Venous Thromboembolism/Pulmonary Embolism Coexist

Active tuberculosis is increasingly recognized not only as a pulmonary infection but also as a systemic inflammatory condition associated with an increased risk of venous thromboembolism, including pulmonary embolism. Epidemiological studies and meta-analyses have reported a higher frequency of thromboembolic events in patients with active tuberculosis compared with non-tuberculous populations, and hospital-based data suggest that this association may be linked to worse clinical outcomes and increased mortality [10,11,12].

The present cases are consistent with this reported association. They illustrate two clinically relevant situations: pulmonary embolism detected at the time of tuberculosis diagnosis and pulmonary embolism developing during the early intensive phase of antituberculous therapy [13,14,15]. Therefore, pulmonary embolism should be considered in patients with active tuberculosis who develop unexplained respiratory deterioration, persistent dyspnea, tachycardia, hypoxemia, rising D-dimer levels, or delayed clinical recovery [16,17].

Smoking may have acted as an additional proinflammatory and prothrombotic cofactor, particularly in the second patient, who had a documented 18 pack-year smoking history. However, in these cases, the central mechanism remains active tuberculosis-related thrombo-inflammation, with smoking considered a possible aggravating factor rather than the sole cause of thrombosis.

### 3.2. Why Tuberculosis Creates a Hypercoagulable State

Active tuberculosis may create a hypercoagulable state through the interaction of systemic inflammation, endothelial activation, platelet stimulation, impaired fibrinolysis, and transient reduction in natural anticoagulant pathways [18,19]. Proinflammatory cytokines such as interleukin-1, interleukin-6, and tumor necrosis factor-alpha promote tissue factor expression, fibrinogen synthesis, thrombin generation, and endothelial dysfunction, all of which favor thrombus formation [20].

These changes are accompanied by a marked imbalance in hemostasis, including increased fibrinogen, factor VIII, D-dimer, and fibrin degradation products, together with decreased levels of key endogenous anticoagulants such as antithrombin III, protein C, and free protein S, thereby removing important physiological restraints on thrombin generation. These alterations create a sustained prothrombotic milieu that provides a strong biological explanation for the increased risk of venous thromboembolism, pulmonary embolism, stroke, and other thrombotic complications observed in patients with active tuberculosis [20,21,22]. This biological mechanism was reflected in both of our patients. Both cases showed reactive thrombocytosis, elevated inflammatory markers, increased D-dimer and fibrinogen levels, and reduced protein C and protein S activity. These abnormalities improved after antituberculous therapy and anticoagulation, supporting an acquired infection-related hypercoagulable state rather than a fixed inherited thrombophilia.

### 3.3. Temporal Pattern of Pulmonary Embolism During the Course of Tuberculosis

Pulmonary embolism associated with active tuberculosis may occur at different points in the disease course. It may be detected at the time of tuberculosis diagnosis, as in our first case, or may develop during the early phase of antituberculous therapy, as in our second case. This temporal variability suggests that thrombotic risk is not limited to the pretreatment period but may persist after treatment initiation while systemic inflammation and coagulation activation remain high.

The two cases therefore illustrate complementary clinical patterns. In Case 1, pulmonary embolism was diagnosed concurrently with active pulmonary tuberculosis. In Case 2, bilateral pulmonary embolism developed during the intensive phase of therapy, in the context of severe pulmonary disease, persistent inflammation, and rising D-dimer levels. This supports the need for continued clinical vigilance during early treatment, particularly in patients with extensive, cavitary, miliary, or extrapulmonary tuberculosis.

Clinically, pulmonary embolism should be suspected when patients with active tuberculosis develop new or worsening dyspnea, tachycardia, hypoxemia, persistent inflammatory activity, rising D-dimer levels, or slower-than-expected recovery [23,24,25]. In such cases, diagnostic imaging should be considered early, even if antituberculous therapy has already been started [26,27].

### 3.4. Biological Markers of TB-Associated Hypercoagulability

Patients with active tuberculosis may show laboratory evidence of systemic inflammation and hypercoagulability, including thrombocytosis, elevated D-dimer and fibrinogen levels, and reduced activity of natural anticoagulants such as protein C and protein S [18,28]. These abnormalities are thought to reflect infection-related thrombo-inflammation and may improve after effective antituberculous therapy [29,30,31,32].

Both patients in the present report showed this pattern at the time pulmonary embolism was diagnosed. They had elevated inflammatory markers, marked reactive thrombocytosis, increased D-dimer and fibrinogen levels, and reduced protein C and protein S activity. At six-month follow-up, these abnormalities had normalized together with clinical and radiological improvement, supporting the interpretation of a reversible, acquired hypercoagulable state related to active tuberculosis.

However, these biomarkers should not be presented as validated predictors of pulmonary embolism. Their role in clinical decision-making remains uncertain. In this report, they should be interpreted as supportive findings that increased suspicion for TB-associated hypercoagulability, rather than as definitive diagnostic or prognostic markers. Larger prospective studies are needed to determine whether coagulation profiling can guide screening, risk stratification, or anticoagulation duration in active tuberculosis [33].

### 3.5. Therapeutic Management in Tuberculosis-Associated Pulmonary Embolism

The coexistence of active tuberculosis and pulmonary embolism requires individualized management, particularly regarding anticoagulant choice, treatment duration, bleeding risk, and interactions with rifampicin-based regimens. In general, TB-associated PE should be treated as provoked venous thromboembolism, alongside full antituberculous therapy [23,26,27]. Reported cases commonly use initial parenteral anticoagulation followed by oral therapy. However, rifampicin complicates anticoagulant selection by inducing hepatic enzymes and P-glycoprotein, which may reduce exposure to oral anticoagulants. Therefore, LMWH, fondaparinux, monitored vitamin K antagonist therapy, or rifabutin substitution may be considered in selected patients [24,34]. The optimal duration of anticoagulant therapy remains uncertain, as no TB-specific trials are available; although most reviews support treating these events as provoked VTE with at least 3 months of anticoagulation, persistent inflammation and ongoing prothrombotic activity in severe or extensive disease argue for a more individualized strategy [35]. The hypercoagulable state observed in both patients appears to be multifactorial. Persistent systemic inflammation likely played a central role through cytokine-driven endothelial dysfunction, increased expression of tissue factor, and enhanced platelet activation [36,37]. Both patients exhibited marked reactive thrombocytosis, with platelet counts exceeding 600,000/μL, reflecting intense inflammatory stimulation and contributing to thrombus propagation.

A particularly important finding in both cases was the transient reduction in protein C and protein S activity, which normalized after antituberculous therapy and anticoagulation [8]. This reversible pattern strongly supports an acquired rather than inherited thrombophilic mechanism, directly related to active tuberculosis. The depletion of these natural anticoagulant pathways may result from inflammatory consumption, hepatic reprioritization of protein synthesis during the acute-phase response, endothelial dysfunction, and severe hypoalbuminemia, especially in the second case [8].

The second case further expands the thrombo-inflammatory spectrum of tuberculosis by illustrating multisystem involvement, where severe pulmonary disease coexisted with intestinal tuberculosis and abdominal ischemic complications. An important observation was the delayed diagnosis of pulmonary embolism in the second patient despite prompt initiation of antituberculous therapy, suggesting that thrombotic risk may persist during the early treatment phase while inflammatory activity remains high. In this setting, persistent dyspnea, tachycardia, rising D-dimer levels, or slower-than-expected clinical recovery should prompt consideration of pulmonary embolism.

In both patients, the normalization of protein C and protein S activity after combined antituberculous treatment and anticoagulation supports an acquired, infection-related hypercoagulable state. Although these findings raise the possibility that such parameters may reflect disease activity and thrombotic risk, this interpretation remains preliminary and requires confirmation in prospective studies [38].

The duration of anticoagulation in tuberculosis-associated pulmonary embolism remains insufficiently defined. At present, management should be guided primarily by standard principles for provoked venous thromboembolism while taking into account the severity of tuberculosis, clinical evolution, and the resolution of inflammatory and coagulation abnormalities, intestinal tuberculosis and abdominal ischemic complications [39,40].

## 4. Clinical Take-Home Messages

Pulmonary embolism should be considered in patients with active tuberculosis who develop unexplained respiratory deterioration, persistent tachycardia, rising D-dimer levels, or delayed clinical improvement, including during the early phase of antituberculous therapy. Reactive thrombocytosis, elevated inflammatory markers, and reduced protein C and protein S activity may accompany tuberculosis-associated hypercoagulability and appeared to improve with treatment in both cases, although their clinical role requires further validation [8].

## 5. Limitations

This case report series should be interpreted within the usual limitations of observational, non-controlled reports. The small sample size precludes generalization, and the proposed mechanisms of hypercoagulability remain exploratory. Although neither patient had a known personal or family history of thromboembolism, a complete inherited thrombophilia evaluation was not available at diagnosis, so an underlying predisposition cannot be fully excluded. In addition, the six-month values presented in Table 1 should be regarded as representative of clinical recovery rather than strict serial measurements, given the retrospective nature of data collection. Finally, the clinical heterogeneity of the two cases limits direct comparison while also reflecting the diversity of thromboembolic presentations that may occur in active tuberculosis. In both cases, pulmonary arterial enhancement was suboptimal, probably related to contrast acquisition timing, and this made the thrombi less sharply demarcated on the reproduced images. Nevertheless, the filling defects were interpreted in the full clinical and radiological context by the treating team and were consistent with pulmonary embolism. This technical limitation should be considered when interpreting the imaging findings.

## 6. Conclusions

These two cases do not establish a new association between tuberculosis and pulmonary embolism, but they illustrate clinically relevant diagnostic scenarios in which pulmonary embolism occurred at presentation and during early antituberculous therapy. Both patients showed a reversible inflammatory and prothrombotic profile, including thrombocytosis, elevated D-dimer and fibrinogen levels, and reduced protein C and protein S activity. These findings support the need for diagnostic vigilance in patients with active tuberculosis who develop unexplained deterioration, persistent dyspnea, rising D-dimer levels, or delayed recovery. Larger prospective studies are needed to determine whether coagulation profiling can support risk stratification or guide management in this setting.

## Figures and Tables

**Figure 1:**
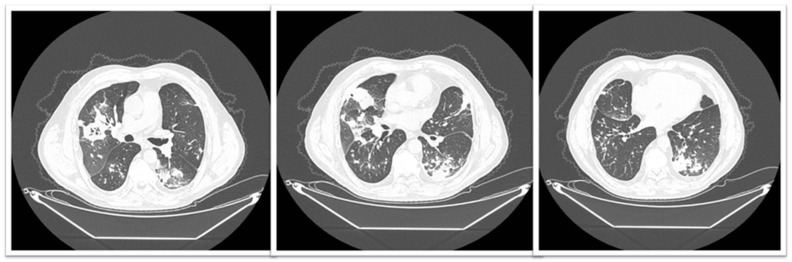
Chest CT imaging showing: centrilobular nodules and bilateral upper-lobe cavitary lesions consistent with active pulmonary tuberculosis.

**Figure 2:**
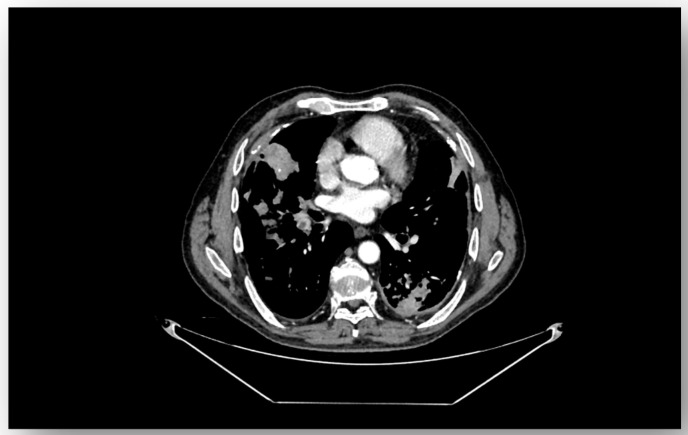
CT pulmonary angiography showing a segmental filling defect in a branch of the right lower pulmonary artery, interpreted as acute pulmonary embolism. Pulmonary arterial enhancement was suboptimal, which limited thrombus conspicuity on the reproduced image.

**Figure 3:**
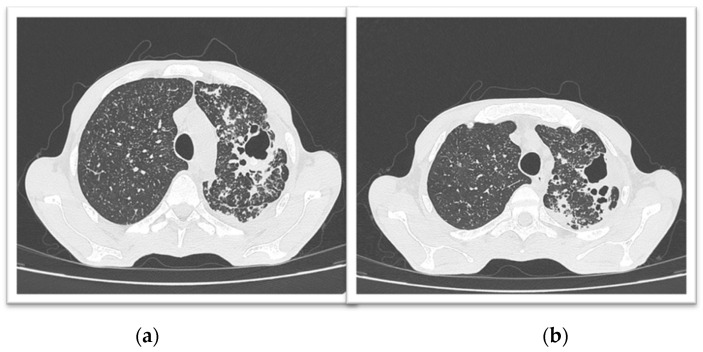
Chest CT imaging showing diffuse bilateral micronodules with miliary distribution, tree-in-bud pattern with confluent consolidation areas (**a**), bronchial ectasia, and multiple cavitary lesions (**b**) in the left upper lobe (largest: 38 × 30 mm), consistent with severe active pulmonary tuberculosis.

**Figure 4:**
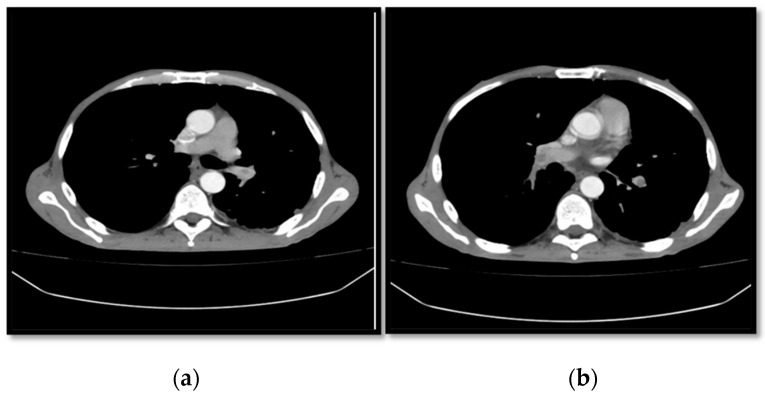
CT pulmonary angiography showing bilateral pulmonary arterial filling defects, interpreted as pulmonary embolism, involving lobar and segmental branches (**a**). Pulmonary arterial enhancement was suboptimal (**b**), which limited thrombus conspicuity on the reproduced image.

**Figure 5:**
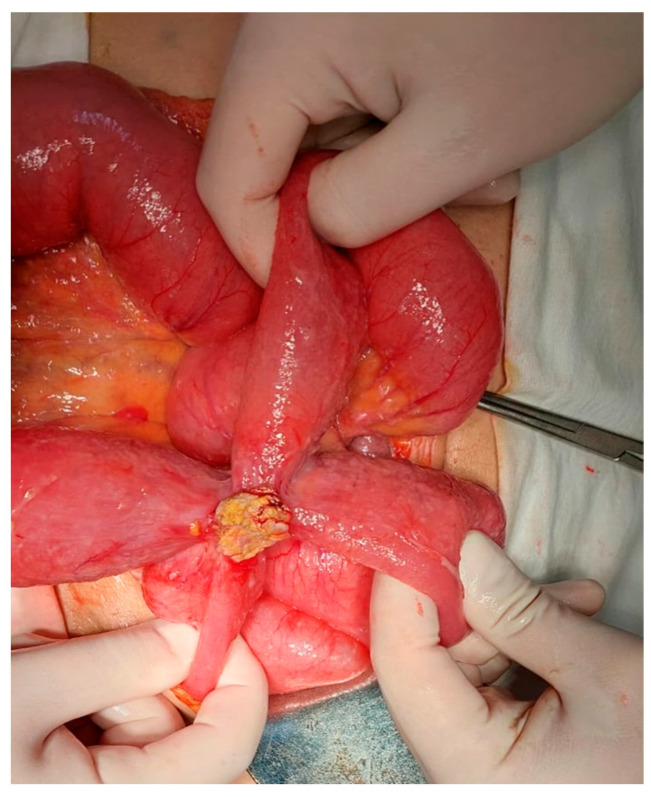
Macroscopic intraoperative examination revealing intestinal loops with congested serosa and an exophytic yellowish-white nodular lesion with caseous center, consistent with intestinal tuberculosis.

**Figure 6:**
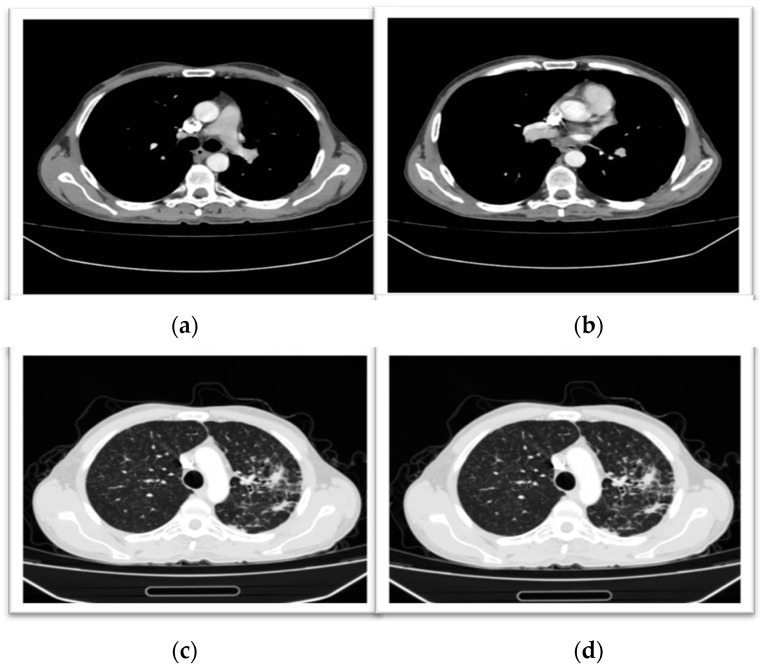
Chest CT at 6 months of antituberculous treatment showing complete resolution of pulmonary embolism with marked regression of pulmonary lesions: mediastinal window (**a**,**b**); lung window (**c**,**d**).

**Table 1:** Paraclinical parameters.

Parameter	Case 1 (Initial)	Case 1 (6 Months)	Normal Range	Case 2 (Initial)	Case 2 (6 Months)	Normal Range
Hemoglobin (g/dL)	10.5	13.8	13–17	9.8	13.2	13–17
Platelets (/μL)	620,000	275,000	150,000–400,000	750,000	240,000	150,000–400,000
White Blood Cells (/μL)	7800	6900	4000–11,000	8100	7200	4000–11,000
C-Reactive Protein (mg/L)	120	3	<5	99	4	<5
Erythrocyte Sedimentation Rate (mm/h)	70	12	<20	90	15	<20
D-dimer (ng/mL)	970	290	<500	2500	340	<500
Protein C activity (%)	50	88	70–140	48	82	70–140
Protein S activity (%)	45	76	60–130	42	73	60–130
Fibrinogen (mg/dL)	620	280	200–400	890	265	200–400
Albumin (g/dL)	3.4	4.1	3.5–5.0	2.5	3.9	3.5–5.0
HIV Serology	Negative	—	–	Negative	—	–

Six-month values reflect documented normalization at follow-up and are presented as representative end-of-treatment values extracted retrospectively.

## Data Availability

The data presented in this study are available upon request from the corresponding author. The data are not publicly available due to the patient’s personal data protection policy of the University and Hospital.
